# The individual and combined effects of air pollution mixtures on the risk of cardiovascular diseases in patients with Cardiovascular-Kidney-Metabolic syndrome at stages 0–3

**DOI:** 10.1371/journal.pone.0346949

**Published:** 2026-06-26

**Authors:** Fengjiao Han, Haiyang Guo, Yang Zheng

**Affiliations:** The First Hospital of Jilin University, Changchun, China; Charite Universitatsmedizin Berlin, GERMANY

## Abstract

**Background:**

This study aims to employ a prospective cohort design to quantitatively assess the association between exposure levels of common ambient air pollutants and the risk of cardiovascular disease (CVD) in patients across stages 0–3 of Cardiovascular-kidney-metabolic (CKM) syndrome. By doing so, it addresses a critical knowledge gap in environmental exposure research within this specific clinical context.

**Methods:**

We analyzed baseline differences between CVD cases/controls using descriptive statistics, parametric/nonparametric tests, and Pearson correlations for air pollutants (PM₁, PM2.5, PM₁₀, NO₂, O₃). Cox regression (Models 1–3, adjusting for sociodemographic/behavioral factors) and RCS assessed pollutant-CVD associations. WQS/qgcomp models evaluated mixture effects via weighted indices and directional weighting. Sensitivity analyses used BKMR, WQS-CVD exposure-response curves, and 2-year lag to address reverse causality.

**Results:**

In single-pollutant analyses, per-interquartile range (IQR) increases in PM₁, PM₂.₅, PM₁₀, and NO₂ were associated with 30% (HR = 1.30, 95% CI 1.17–1.45), 35% (HR = 1.35, 95% CI 1.21–1.51), 52% (HR = 1.52, 95% CI 1.35–1.70), and 30% (HR = 1.30, 95% CI 1.17–1.45) elevations in CVD risk, respectively. No significant association was found for O₃. In mixture analyses, all three quantile g-computation (qgcomp) models linked combined pollutant exposure to significantly higher CVD risk (Model 1: HR = 1.10, 95% CI 1.04–1.17; Model 2: HR = 1.11, 95% CI 1.04–1.18; Model 3: HR = 1.12, 95% CI 1.05–1.19). PM₁₀ emerged as the dominant driver of the mixture effect.

**Conclusion:**

Higher exposure levels to ambient air pollutants are associated with an increased risk of cardiovascular disease in patients with Stage 0–3 CKM syndrome.

## Introduction

Air pollution occurs when pollutant concentrations surpass environmental self-purification capacity, threatening human health via atmospheric media. As early as 2017, ambient particulate matter pollution, along with high systolic blood pressure, tobacco use, and a high-sodium diet, had emerged as the four leading risk factors contributing to deaths and disability-adjusted life years (DALYs) among Chinese residents, drawing widespread attention from the Chinese government [1]. The 2021 Global Burden of Disease (GBD) study further solidified air pollution’s position as a global health threat. Data indicate that particulate matter pollution (encompassing both ambient and household air pollution) ranked as the third-highest risk factor globally in 2021, accounting for 8.0% (95% confidence interval [CI]: 6.7%−9.4%) of total DALYs [2]. Air pollution has undoubtedly become one of the most severe global environmental issues, posing a significant threat to human health. Numerous studies have established a strong correlation between air pollution and the onset and progression of various diseases, particularly cardiovascular diseases [3–5], respiratory diseases [6,7], and mental disorders [8,9].

Existing epidemiological evidence suggests that the association between exposure to air pollutants and cardiovascular diseases (CVD) is the most pronounced. A nationwide cohort study conducted by Chinese scholars has confirmed that long-term exposure to ambient ozone increases the risk of cardiovascular diseases [10]. Another Mendelian randomization study has similarly unveiled the link between the two [11]. Regarding the pathophysiological mechanisms underlying this association, current research primarily focuses on oxidative stress [12] and inflammatory responses [13,14], The interplay between these two factors damages vascular endothelial cells, leading to impaired endothelial-dependent vasodilation [15]. Simultaneously, abnormal hemorheological parameters, such as elevated fibrinogen levels, increase blood viscosity, thereby promoting thrombus formation [16].

Based on the interactive pathophysiological mechanisms among metabolic risk factors, chronic kidney disease (CKD), and the cardiovascular system, the American Heart Association (AHA) officially proposed the disease classification criteria for Cardiovascular-Kidney-Metabolic Syndrome (CKM syndrome) on October 9, 2023 [17]. CKM stage 0 is characterized by the absence of cardiovascular disease (CVD) risk factors, with prevention primarily focused on minimizing the risk of CKD or CVD development. CKM stage 1 presents as overweight/obesity, requiring interventions to address adiposity and prevent progression of metabolic risk factors. CKM stage 2 involves coexisting metabolic risk factors and renal lesions, necessitating integrated management of metabolic abnormalities and CKD to block transition to subclinical/clinical CVD. CKM stage 3 comprises patients with subclinical CVD, very high-risk CKD, and/or high predicted CVD risk, with the core objective of preventing progression to clinical CVD and renal failure. Compared to individuals at stage 0 of CKM syndrome, those at stages 1–4 exhibited a 1.24-fold, 1.72-fold, 2.58-fold, and 3.73-fold increase in the risk of all-cause mortality, respectively [18].The greatest clinical impact of CKM syndrome on morbidity and mortality stems from its substantial burden of CVD [17]. Underestimation of CVD risk may lead to inadequate preventive measures and delayed therapeutic interventions, thereby worsening patient prognosis [19]. Conversely, early systematic intervention across CKM stages 0–3 can significantly reduce the incidence and progression of clinical CVD. Given these risk stratification characteristics, establishing an early warning system for CKM syndrome holds significant clinical value in improving the prognosis of patients with this condition.

Previous studies have predominantly concentrated on evaluating the efficacy of biomarkers in predicting cardiovascular events among patients with CKM syndrome [20–24]. However, the association between air pollution exposure and the progression of CKM disease remains unexplored. This study aims to employ a prospective cohort design to quantitatively analyze the relationship between exposure levels of common ambient air pollutants and the risk of cardiovascular diseases in patients at stages 0–3 of CKM syndrome, thereby filling the research gap in environmental exposure studies within this field.

## Materials and methods

### study population

#### Data source.

The study population was derived from the China Health and Retirement Longitudinal Study (CHARLS), a nationally representative longitudinal survey. The data collection for CHARLS received approval from the Biomedical Ethics Review Committee of Peking University (IRB00001052–11015), and the study protocol adhered to the ethical standards outlined in the 1975 Declaration of Helsinki. All participants in the study provided informed consent after receiving comprehensive written information. The national baseline survey of CHARLS was conducted from June 2011 to March 2012, with follow-ups conducted biennially using face-to-face computer-assisted personal interviews (CAPI). To date, CHARLS has released data from four waves of follow-up (Wave 2 in 2013, Wave 3 in 2015, Wave 4 in 2018, and Wave 5 in 2020) [25].

**Fig 1:**
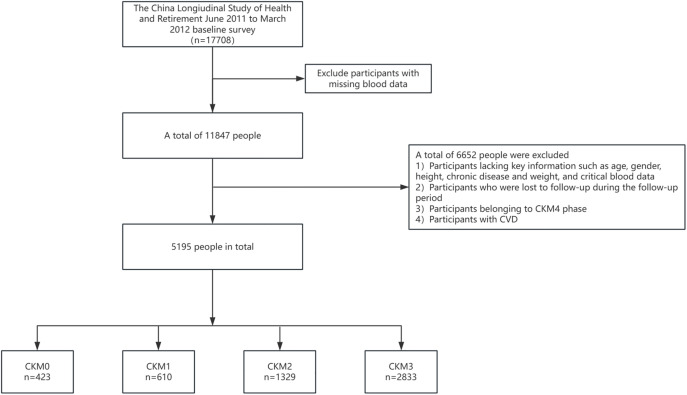
The flowchart.

The flowchart (Fig 1) outlines the inclusion and exclusion criteria for this study. A total of 5,195 participants were ultimately included in the final analysis.

### Definitions of Stage 0 to Stage 3 of Cardiovascular-Kidney-Metabolic (CKM) Syndrome

The classification of Cardiovascular-Kidney-Metabolic (CKM) syndrome stages adheres to the criteria outlined in the American Heart Association (AHA) Presidential Advisory concerning CKM syndrome patients [17]. The descriptions for each stage are as follows: Stage 0: Individuals show no evidence of metabolic abnormalities, cardiovascular lesions, or renal impairment. Stage 1: Features of this stage include overweight/obesity (with a BMI ≥ 25 kg/m² or excessive waist circumference) or isolated adipose tissue dysfunction (without accompanying metabolic risk factors such as hypertension, dyslipidemia, or chronic kidney disease [CKD]). Stage 2: The presence of dyslipidemia(DL) (≥135 mg/dL), hypertension(HTN) (≥130/80 mmHg), metabolic syndrome(MetS), diabetes mellitus(DM), or a confirmed diagnosis of CKD (defined by an estimated glomerular filtration rate [eGFR] < 60 mL/min/1.73m², calculated using the Chinese-modified Modification of Diet in Renal Disease [C-MDRD] equation) characterizes this stage. Stage 3: Beyond the metabolic abnormalities mentioned above, patients in this stage must also present with one or more high-risk characteristics: subclinical cardiovascular disease (CVD), indicated by a 10-year cardiovascular event risk ≥ 10% as predicted by the Framingham Risk Score [26], or CKD progression to stages G4-G5 (eGFR < 30 mL/min/1.73m²) and classified as very high risk according to the Kidney Disease: Improving Global Outcomes (KDIGO) guidelines. The eGFR is determined using the C-MDRD equation [27], and is employed to categorize CKD stages in line with KDIGO criteria [17]. The specific definition of CKM syndrome can be found in S1 Table.

### Assessment of Air Pollution Exposure

The concentrations of air pollutants (PM₁, PM_2.5_, PM₁₀, NO₂, and O₃) were obtained from the China High-resolution Air Pollution (CHAP) dataset. This dataset integrates multi-source environmental monitoring data with multimodal artificial intelligence algorithms to construct a nationwide, spatially resolved database of air pollution exposure. By employing a spatiotemporal fusion model and deep learning architecture, the CHAP dataset generates daily gridded concentration data for PM₁, PM₂.₅, PM₁₀, and O₃ at a spatial resolution of 1 km × 1 km [28]. Due to limitations in satellite retrieval algorithms, the spatial resolution of NO₂ concentration data was optimized to 10 km × 10 km. Following multi-source validation, the CHAP dataset has emerged as an authoritative data source for environmental epidemiological research in China, with its reliability confirmed in multiple studies [29–31].

To account for the lagged health effects of air pollutants, this study utilized spatial interpolation algorithms to integrate the CHAP raster data with geocoded community-level resident information from the CHARLS Primary Sampling Unit (PSU) dataset. This integration enabled the assignment of annual average pollutant exposure concentrations for three consecutive years (2008–2010) at the city level for each participant.

### follow-up outcomes

This study utilized a prospective follow-up cohort spanning the period from 2011 to 2020 as the observation population, with the cumulative incidence of composite cardiovascular disease (CVD) events serving as the outcome measure.

Information on a history of heart disease was obtained through a standardized question: “Has a doctor ever told you that you have been diagnosed with a heart attack, including myocardial infarction, coronary heart disease, angina pectoris, congestive heart failure, and other types of heart diseases?” The occurrence of stroke was determined using the following question: “Has a doctor ever told you that you have been diagnosed with a stroke?” Cardiovascular disease (CVD) was defined as self-reported heart disease or stroke. The disease onset time refers to the duration from the baseline survey date to the date when the participant experienced the disease, provided that the disease event was adequately documented. In cases where precise temporal data were unavailable, we reasonably estimated the disease onset time based on the median interval between the date of the first interview and the wave in which the disease information was recorded. This recording method has been validated in multiple previous studies [32–34].

### Covariates

Confounding variables were selected based on prior studies [30,31] and included the following categories: [1] Demographic factors: age, gender, place of residence, and marital status; [2] Health-related behaviors: smoking status, alcohol consumption, and sleep disorders; [3] Socioeconomic status: total per capita household consumption, educational attainment, and type of cooking fuel.

### Statistical analysis

First, descriptive statistics were conducted to analyze the baseline characteristics of the incident cardiovascular disease (CVD) group and the control group. For continuous variables, parametric tests (t-test/analysis of variance, ANOVA) or nonparametric tests (Mann-Whitney U test/Kruskal-Wallis test) were applied to quantify differences in distributions between groups, while the chi-square test was used for categorical variables. A Pearson correlation coefficient matrix was employed to perform pairwise correlation analyses among air pollutants, including PM₁, PM_2.5_, PM₁₀, NO₂, and O₃, to elucidate the synergistic patterns of mixed exposures and identify potential multicollinearity issues.

Further, multivariate cox regression models were employed to examine the associations between air pollutants and the risk of incident cardiovascular disease (CVD). In these models, various potential confounding factors were adjusted based on prior studies. Specifically, Model 1 represented the unadjusted crude model; Model 2 adjusted for sociodemographic characteristics (including age, gender, place of residence, educational attainment, marital status, total per capita household consumption, and type of cooking fuel); and Model 3 further incorporated behavioral health factors (smoking status, alcohol consumption, and sleep disorders) on top of the variables in Model 2. To further elucidate the concentration-response relationships between air pollutants and incident CVD risk, restricted cubic splines (RCS) with three knots were utilized.

Next, given the reality of complex mixtures of air pollutant exposures, we employed two complementary methods—Weighted Quantile Sum (WQS) regression and quantile g-computation (qgcomp)—to comprehensively investigate the overall impact of air pollutant mixture exposure on the risk of cardiovascular disease (CVD) incidence. The WQS model has been previously described in detail [35]. The dataset was randomly split into two subsets: a training subset (40%) and a validation subset (60%). After 50,000 bootstrap iterations, the R package “gWQS” calculated a weighted linear index (ranging from 0 to 1), which reflects the total exposure burden of all air pollutants for each individual. The weights assigned to each air pollutant were proportional to their contributions to the WQS index. To account for directional uncertainty in the mixture components, we further applied the qgcomp method [36]. This model assigns both positive and negative weights to each component in the mixture, ensuring that the absolute sum of weights (regardless of direction) equals 1.0.

To determine whether the relationship between air pollutants and the risk of incident cardiovascular disease is subject to interference from other factors, subgroup analyses were further conducted with respect to factors such as age, gender, HTN, DM, DL, MetS, and CKM staging.

Finally, sensitivity analyses were conducted to enhance the robustness of the study findings. First, the Bayesian Kernel Machine Regression (BKMR) model [37] was employed to further evaluate the individual and joint effects of exposures to multiple air pollutants on the risk of cardiovascular disease (CVD) incidence. Second, exposure-response curves were plotted to illustrate the relationship between the Weighted Quantile Sum (WQS) index and CVD risk. Third, cox regression analyses were repeated after excluding participants with a disease onset time of less than 2 years to assess the potential impact of reverse causation or protopathic bias. Fourth, building upon the original Model 3, a range of crucial clinical cardiovascular-metabolic factors were incorporated, including hypertension, diabetes, hyperlipidemia, CKM staging, metabolic syndrome, body mass index (BMI), waist circumference, low-density lipoprotein cholesterol (LDL-C), high-density lipoprotein cholesterol (HDL-C), total cholesterol (TC), triglycerides (TG), and C-reactive protein (CRP). Subsequently, Cox proportional hazards regression analysis was repeated.

## Results

### Characteristics of study population

This study enrolled 5,195 participants, among whom 1,146 (22.1%) developed incident cardiovascular disease (CVD) after long-term follow-up. Based on CVD incidence status during follow-up, participants were categorized into the case group (n = 1,146) and the control group (n = 4,049). A systematic comparison of baseline characteristics between the two groups, as shown in **Table 1**, revealed statistically significant differences in age, gender, type of cooking fuel, sleep disorders, metabolic diseases (hypertension/diabetes/hyperlipidemia), medication history, smoking and alcohol consumption behaviors, CKM staging, metabolic syndrome, body mass index (BMI), waist circumference, blood pressure levels, and multiple hematological parameters (all P-values < 0.05).

**Table 1:** Baseline characteristics classified by whether cardiovascular disease occurs.

Variable^a^	level	Non-CVD	CVD	p
n		4049	1146	
Age, year		57.16 (8.79)	59.33 (8.47)	<0.001
Gender, n (%)	Female	2222 (54.9)	676 (59.0)	0.015
	Male	1827 (45.1)	470 (41.0)	
Education, n (%)	primary or below	3673 (90.7)	1027 (89.6)	0.278
	second/high school or above	375 (9.3)	119 (10.4)	
Marital, n (%)	married	3709 (91.6)	1029 (89.8)	0.064
	unmarried	340 (8.4)	117 (10.2)	
Lives in rural or urban, n (%)	Urban Community	1263 (31.2)	340 (29.7)	0.342
	Rural Village	2786 (68.8)	806 (70.3)	
Total per capita household consumption, RMB		6005.92 (7062.72)	6203.90 (9323.85)	0.437
Main source of cooking fuel, n (%)	Coal	423 (10.5)	149 (13.0)	0.013
	Natural gas	338 (8.4)	97 (8.5)	
	Marsh gas	79 (2.0)	17 (1.5)	
	Liquefied Petroleum Gas	556 (13.8)	141 (12.3)	
	Electric	724 (17.9)	187 (16.3)	
	Crop residue/Wood burning	1905 (47.1)	542 (47.3)	
	other	18 (0.4)	13 (1.1)	
Sleep disorders, n (%)	Rarely or none of the time <1 day	2114 (52.6)	490 (43.1)	<0.001
	Some or a little of the time 1–2 days	619 (15.4)	193 (17.0)	
	Occasionally or a moderate amount of the time 3–4 days	571 (14.2)	207 (18.2)	
	Most or all of the time 5–7 days	712 (17.7)	246 (21.7)	
HTN, n (%)	no	2761 (68.2)	619 (54.0)	<0.001
	yes	1288 (31.8)	527 (46.0)	
DM, n (%)	no	3561 (87.9)	954 (83.2)	<0.001
	yes	488 (12.1)	192 (16.8)	
DL, n (%)	no	2439 (60.7)	600 (52.7)	<0.001
	yes	1580 (39.3)	539 (47.3)	
Take medicine for diabetes, n (%)	no	3968 (98.0)	1096 (95.6)	<0.001
	yes	81 (2.0)	50 (4.4)	
Take medicine for hypertension, n (%)	no	3579 (88.4)	904 (78.9)	<0.001
	yes	470 (11.6)	242 (21.1)	
Take medicine for dyslipidemia, n (%)	no	4029 (99.5)	1133 (98.9)	0.028
	yes	20 (0.5)	13 (1.1)	
Smoking, n (%)	Ex-smoker	266 (6.6)	95 (8.3)	0.035
	Non-smoker	2564 (63.3)	740 (64.6)	
	smoker	1219 (30.1)	311 (27.1)	
Drinking, n (%)	no	2677 (66.1)	800 (69.8)	0.021
	yes	1372 (33.9)	346 (30.2)	
CKD, n (%)	no	3832 (94.8)	1073 (94.0)	0.281
	yes	209 (5.2)	69 (6.0)	
eGFR		107.98 (28.88)	106.09 (29.14)	0.050
CKM, n (%)	0	359 (8.9)	64 (5.6)	<0.001
	1	498 (12.3)	112 (9.8)	
	2	1046 (25.8)	283 (24.7)	
	3	2146 (53.0)	687 (59.9)	
MetS, n (%)	no	3354 (83.2)	871 (76.2)	<0.001
	yes	677 (16.8)	272 (23.8)	
BMI, kg/m2		23.44 (4.23)	24.25 (4.04)	<0.001
Waist measurement, cm		83.32 (11.94)	86.21 (13.15)	<0.001
Sbp, mmHg		127.03 (20.04)	132.75 (21.50)	<0.001
Dbp, mmHg		74.40 (11.72)	77.27 (12.48)	<0.001
Glycated hemoglobin, mg/dl		5.21 (0.70)	5.35 (0.95)	<0.001
Glucose, mg/dl		107.68 (30.33)	113.28 (43.77)	<0.001
TG, mg/dl		129.93 (109.40)	139.86 (117.91)	0.008
TC, mg/dl		193.19 (38.20)	198.05 (38.99)	<0.001
LDL-C, mg/dl		115.69 (34.16)	120.39 (36.18)	<0.001
Creatinine, mg/dl		0.76 (0.18)	0.76 (0.18)	0.848
Uric acid, mg/dl		4.36 (1.20)	4.35 (1.24)	0.889
HDL-C, mg/dl		52.08 (15.28)	50.29 (15.25)	<0.001
hs-CRP, mg/L		2.33 (6.75)	2.89 (7.29)	0.016

^a^Data were expressed as mean (SD) or n (%). HTN hypertension, DM diabetes, DL dyslipidemia, CKD chronic Kidney Disease, CVD cardiovascular disease, Mets metabolic Syndrome, eGFR Estimated Glomerular Filtration Rate, BMI body mass index, Sbp systolic blood pressure, Dbp diastolic pressure, TG triglyceride, TC total cholesterol, LDL-C low-density lipoprotein cholesterol, hs-CRP hypersensitive C-Reactive Protein, HDL-C high-density lipoprotein cholesterol.

Over the three-year study period, the median (interquartile range, IQR) annual concentrations of PM₁, PM_2.5_, PM₁₀, O₃, and NO₂ were 31.2 (14.1) μg/m³, 56.4 (27.4) μg/m³, 97.3 (48.7) μg/m³, 84.3 (7.5) μg/m³, and 27.3 (15.1) μg/m³, respectively. Pearson correlation coefficients were calculated to systematically evaluate the synergistic association patterns among these five typical air pollutants (S1 Fig). The results revealed strong positive correlations between NO₂ and PM₁ (r = 0.87), PM₁₀ (r = 0.85), and PM_2.5_ (r = 0.83), suggesting significant common sources of traffic-related emissions and particulate matter pollution. PM₁ exhibited extremely high correlations with PM₁₀ (r = 0.92) and PM_2.5_ (r = 0.98), indicating homologous emission characteristics between ultrafine particles (PM₁) and inhalable particulate matter (PM₁₀/ PM_2.5_). The correlation coefficient between PM₁₀ and PM_2.5_ reached 0.95, further confirming the shared origins of coarse and fine particulate matter pollution. Notably, O₃ showed weaker correlations with other pollutants (r = 0.23–0.53), suggesting that ozone pollution is primarily driven by photochemical reactions and exhibits relatively independent synergistic effects compared to particulate matter and nitrogen oxide pollution.

### Associations between Individual Air Pollutants and the Risk of Incident Cardiovascular Disease in Patients with Stage 0–3 CKM Syndrome

First, we constructed three Cox proportional hazards models based on the aforementioned baseline characteristics. Model 1 was an unadjusted crude model; Model 2 adjusted for sociodemographic characteristics (including age, gender, place of residence, educational attainment, marital status, total per capita household consumption, and type of cooking fuel); and Model 3 further incorporated behavioral health factors (smoking, alcohol consumption, and sleep disorders) on top of the variables in Model 2. Using the annual average concentration of each individual air pollutant as the exposure metric, the results presented in **Table 2** demonstrate that, in the fully adjusted model, each interquartile range (IQR) increase in the concentrations of PM₁, PM_2.5_, PM₁₀, and NO₂ was associated with a 30% (HR = 1.30, 95% CI 1.17–1.45), 35% (HR = 1.35, 95% CI 1.21–1.51), 52% (HR = 1.52, 95% CI 1.35–1.70), and 30% (HR = 1.30, 95% CI 1.17–1.45) elevation in the risk of cardiovascular disease (CVD) incidence, respectively. Quantitative comparison revealed that PM₁₀ exhibited the highest magnitude of effect among all evaluated pollutants. In contrast, no significant association was observed between O₃ concentration and CVD risk.

**Table 2:** Connection between one air pollutant and CVD risk in CKM syndrome’s stage 0-3 patients.

Air pollutant (IQR)	Model 1^a^		Model 2^b^		Model 3^c^	
	HR(95%CI)	P value	HR (95 % CI)	P value	HR (95 % CI)	P value
NO_2_(15.1 μg/m³)	1.26(1.13,1.39)	<0.001	1.28(1.15,1.43)	<0.001	1.30(1.17,1.45)	<0.001
O_3_(7.5 μg/m³)	0.97(0.89,1.06)	0.500	0.98(0.90,1.07)	0.622	0.99(0.91,1.08)	0.815
PM_1_(14.1 μg/m³)	1.28(1.15,1.41)	<0.001	1.28(1.16,1.43)	<0.001	1.30(1.17,1.45)	<0.001
PM_2.5_(27.4 μg/m³)	1.33(1.19,1.47)	<0.001	1.33(1.19,1.48)	<0.001	1.35(1.21,1.51)	<0.001
PM_10_(48.7 μg/m³)	1.47(1.32,1.64)	<0.001	1.50(1.34,1.68)	<0.001	1.52(1.35,1.70)	<0.001

^a^Model 1 represented the unadjusted crude model.

^b^Model 2 adjusted for sociodemographic characteristics (including age, gender, place of residence, educational attainment, marital status, total per capita household consumption, and type of cooking fuel).

^c^Model 3 further incorporated behavioral health factors (smoking status, alcohol consumption, and sleep disorders) on top of the variables in Model 2

**Fig 2:**
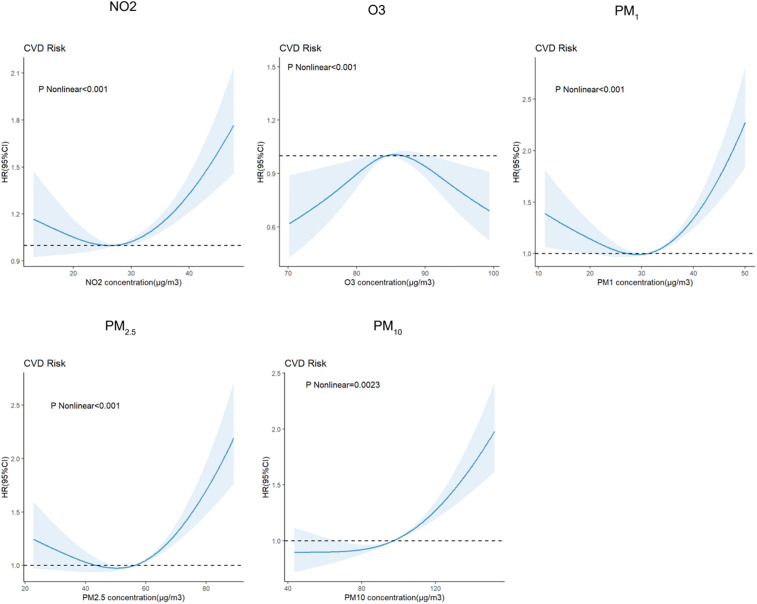
Analyzing annual average air pollutant exposure and CVD risk with Restrictive cubic splines.

Subsequently, to clarify the nonlinear exposure-response relationship, we employed restricted cubic spline (RCS) curves for further analysis (Fig 2). The results revealed significant nonlinear associations between the concentrations of PM₁, PM_2.5_, PM₁₀, NO₂, and O₃ and the risk of cardiovascular disease (CVD) incidence (all P nonlinear < 0.001).

### Associations between Combined Air Pollutant Exposure and the Risk of Incident Cardiovascular Disease in Patients with Stage 0–3 CKM Syndrome

**Fig 3:**
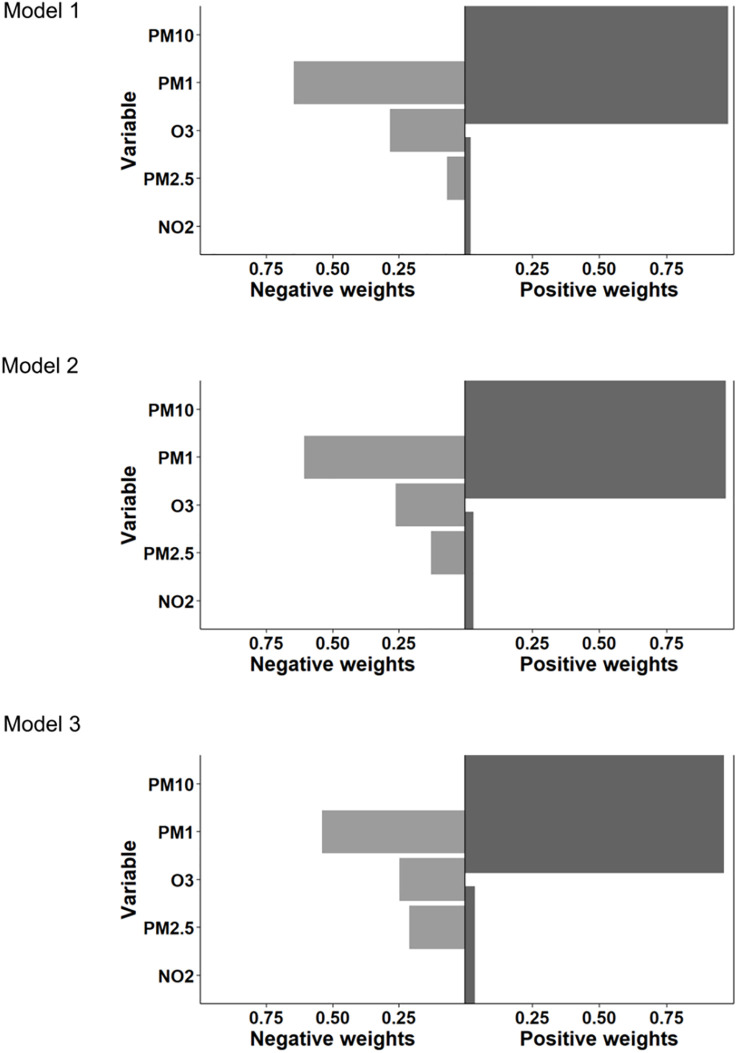
Estimate the weight of five air pollutants used for cardiovascular disease risk using a qgcomp.

**Fig 4:**
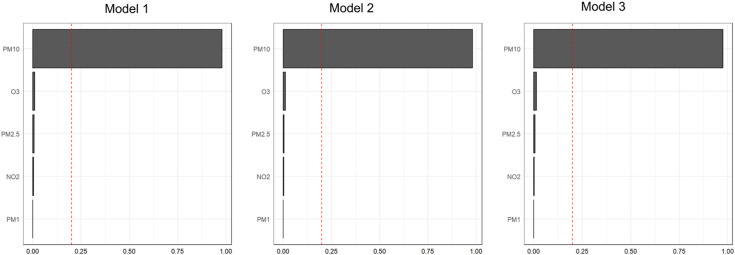
Estimate the weight of five air pollutants used for cardiovascular disease risk using a WQS.

This study employed both the Weighted Quantile Sum (WQS) regression model and the quantile g-computation (qgcomp) model to comprehensively evaluate the joint exposure effects of air pollutant mixtures on the risk of cardiovascular disease (CVD). Both models utilized a hierarchical modeling strategy analogous to cox regression, with Model 1 being an unadjusted crude model, Model 2 adjusting for sociodemographic characteristics, and Model 3 further incorporating behavioral health factors. The results presented in **Table 3** demonstrate consistent findings across the three qgcomp models: mixed pollutant exposure was significantly associated with an elevated risk of CVD (Model 1: HR = 1.10, 95% CI 1.04–1.17; Model 2: HR = 1.11, 95% CI 1.04–1.18; Model 3: HR = 1.12, 95% CI 1.05–1.19). Fig 3 illustrates the pollutant weight estimates from qgcomp, identifying PM₁₀ as the strongest positive driver (with the highest weight coefficient), followed by NO₂. Similarly, the three WQS regression models yielded highly consistent results (Model 1: HR = 1.07, 95% CI 1.04–1.10; Model 2: HR = 1.06, 95% CI 1.02–1.10; Model 3: HR = 1.06, 95% CI 1.03–1.10). Fig 4 depicts the pollutant weight distribution within the WQS index, further confirming that PM₁₀ contributed the most to the mixed exposure, suggesting that particulate matter pollution (particularly PM₁₀) is the primary component driving the CVD risk associated with air pollutant mixtures. The concordance between the two models provides robust evidence for attributing health risks to complex ambient air pollution exposures.

**Table 3:** Link between combined air pollutants and CVD risk in CKM syndrome’s stage 0-3 patients.

	Qgcomp	WQS
	HR(95%CI)	HR(95%CI)
Model 1^a^	1.10(1.04,1.17)	1.07(1.04,1.10)
Model 2^b^	1.11(1.04,1.18)	1.06(1.02,1.10)
Model 3^c^	1.12(1.05,1.19)	1.06(1.03,1.10)

^a^Model 1 represented the unadjusted crude model.

^b^Model 2 adjusted for sociodemographic characteristics (including age, gender, place of residence, educational attainment, marital status, total per capita household consumption, and type of cooking fuel).

^c^Model 3 further incorporated behavioral health factors (smoking status, alcohol consumption, and sleep disorders) on top of the variables in Model 2

### Subgroup analysis

To conduct an in – depth analysis of whether the association between exposure to different air pollutants and the risk of incident cardiovascular disease in patients with CKM syndrome stages 0–3 is subject to interference from other potential factors, this study carried out subgroup analyses focusing on key variables such as age, gender, HTN, DM, DL, MetS, and CKM staging.

**Fig 5:**
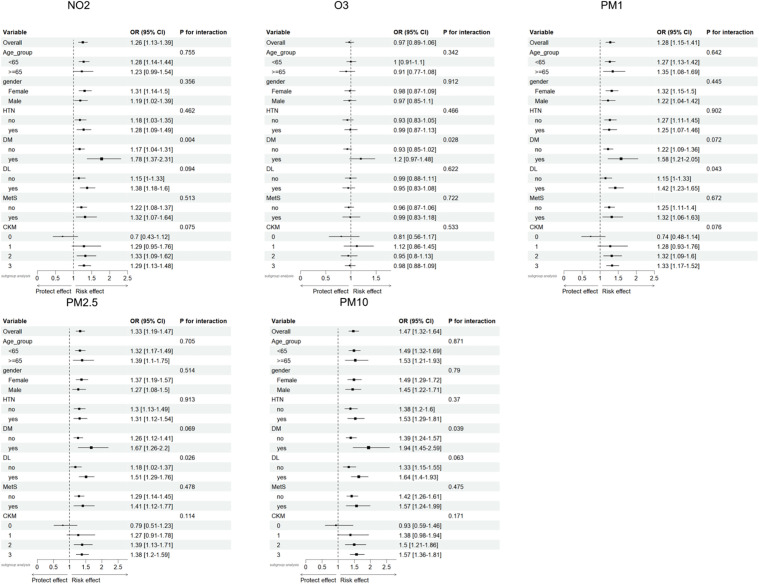
Subgroup analysis.

Fig 5 presents the results of the stratified analyses for different air pollutants. When analyzing NO₂, a significant difference was observed in the diabetes subgroup. Specifically, compared with non – DM patients, DM patients were more prominently affected (HR for non – DM patients = 1.17, 95% CI: 1.04–1.31; HR for DM patients = 1.78, 95% CI: 1.37–2.31, with P for interaction = 0.004). However, no statistically significant interactions were observed in different subgroups divided according to other factors.

When analyzing O₃, a significant difference was also found in the diabetes subgroup. Compared with non – DM patients, DM patients were more significantly affected (HR for non – DM patients = 0.93, 95% CI: 0.85–1.02; HR for DM patients = 1.20, 95% CI: 0.97–1.48, with P for interaction = 0.028). For different subgroups formed based on other factors, no obvious interactions were detected.

In the analysis of PM₁, a significant difference was observed in the DL subgroup. Compared with non – DL patients, DL patients were more markedly affected (HR for non – DL patients = 1.15, 95% CI: 1.00–1.33; HR for DL patients = 1.42, 95% CI: 1.23–1.65, with P for interaction = 0.043). No significant interactions were observed in different subgroups of other factors.

When analyzing PM_2.5_, a significant difference was also present in the DL subgroup. Compared with non – DL patients, DL patients were more significantly affected (HR for non – DL patients = 1.18, 95% CI: 1.02–1.37; HR for DL patients = 1.51, 95% CI: 1.29–1.76, with P for interaction = 0.026). For different subgroups divided according to other factors, no obvious interactions were found.

In the analysis of PM₁₀, a significant difference was observed in the diabetes subgroup. Compared with non – DM patients, DM patients were more prominently affected (HR for non – DM patients = 1.39, 95% CI: 1.24–1.57; HR for DM patients = 1.94, 95% CI: 1.45–2.59, with P for interaction = 0.039). No obvious interactions were observed in different subgroups of other factors.

### Sensitivity Analysis

To validate the robustness of our study conclusions, we conducted multiple sensitivity analyses to reinforce the reliability of the evidence chain: Firstly, Bayesian Kernel Machine Regression (BKMR) Model Validation: We assessed the joint exposure effects of air pollutant mixtures using the BKMR model (S2 Fig 3). The results revealed a highly consistent pattern of synergistic interactions between pollutants such as PM₁₀ and NO₂ on CVD risk compared to the WQS and qgcomp models, confirming the methodological validity of our mixture analysis approach. Secondly, Exposure-Response Relationship Verification via WQS Index: We examined the exposure-response relationship between the weighted quantile sum (WQS) index and CVD incidence risk (S3 Fig). No significant deviation from the primary analysis results was observed, further supporting the stability of the combined exposure effects. Thirdly, Cox Regression Replication with Exclusion of Short-Term Cases: To minimize potential reverse causality bias, we excluded patients with CVD onset within 2 years of exposure assessment and repeated the cox regression analysis (S2 Table). The resulting hazard ratios (HRs) and confidence intervals (CIs) largely overlapped with those of the primary model, providing additional evidence for the robustness of our findings. Fourth, after adding confounding factors such as HTN, DM, DL, CKM staging, MetS, BMI, waist circumference, LDL-C, HDL-C, TC, TG, and CRP into Model 3, Cox regression analysis was conducted again. The results are shown in S3 Table. After introducing these variables, although the association between air pollution exposure and the risk of cardiovascular diseases weakened to a certain extent, it still maintained statistical significance. This also confirms the robustness of our findings.

## Discussion

This study, based on a prospective cohort of 5,195 participants, found that air pollutants (including PM₁, PM_2.5_, PM₁₀, NO₂, and O₃) may accelerate the progression of clinical cardiovascular disease (CVD) incidence in patients with stage 0–3 cardiometabolic-kidney-metabolic (CKM) syndrome. Single-pollutant cox regression analyses revealed independent positive associations between PM₁, PM_2.5_, PM₁₀, and NO₂ and the incidence of CVD, whereas no statistically significant association was observed between O₃ and CVD risk. Further analysis using multi-pollutant quantile g-computation (qgcomp) and weighted quantile sum (WQS) models confirmed a positive correlation between combined air pollutant exposure and CVD risk. Notably, PM₁₀ exhibited the strongest independent effect among individual pollutants and maintained dominant predictive power in multipollutant models. Sensitivity analyses validated the robustness of these core findings.

The mechanisms by which air pollutants induce and exacerbate cardiovascular disease (CVD) are widely attributed to two core pathways in current scientific literature: oxidative stress and inflammatory responses [38]. After deposition in the alveoli, air pollutant particles are phagocytosed by alveolar macrophages, triggering an inflammatory cascade. This process stimulates macrophages to release pro-inflammatory cytokines (e.g., TNF-α, IL-6) [39], thereby initiating both localized pulmonary and systemic inflammatory responses [40]. Notably, certain ultrafine particles can translocate directly into the bloodstream via the alveolo-capillary barrier [41], leading to a marked elevation in circulating levels of free radicals and reactive oxygen/nitrogen species (ROS/RNS). This surge induces systemic oxidative stress [42], accompanied by aberrant increases in fibrinogen concentration and plasma viscosity, which promote hypercoagulability and thrombotic tendencies [43]. Concurrently, dysregulation of the autonomic nervous system-sympathetic nervous system axis impairs both endothelial-dependent (e.g., NO-mediated) and -independent vasodilation [38], ultimately establishing the pathophysiological basis for cardiovascular injury.

Excessive adipose tissue hyperplasia and dysfunction constitute one of the core pathophysiological hallmarks of CKM syndrome [17], with perivascular adipose tissue (PVAT) exerting a dual role in both vascular homeostasis maintenance and pathological transformation [44]. Under physiological conditions, PVAT regulates vascular tone equilibrium through mechanical cushioning effects and paracrine signaling [45]. Its secretion of vasoactive factors (e.g., adiponectin, nitric oxide [NO], and peptides of the renin-angiotensin system) collectively sustains vasodilatory function. However, during obesity-associated insulin resistance, PVAT undergoes significant activation of oxidative stress and chronic low-grade inflammation [46], triggering a remodeling of its vasoactive factor expression profile: adiponectin and NO biosynthesis are suppressed, while the expression of vasoconstrictive mediators such as angiotensin II (Ang II) is upregulated [45]. This pathogenic cascade is primarily driven by a pro-inflammatory adipokine network, characterized by aberrant secretion of cytokines including TNF-α, IL-6, and IL-8. These mediators induce endothelial insulin signaling dysfunction, disrupt calcium homeostasis in vascular smooth muscle cells, and promote extracellular matrix remodeling, culminating in impaired vasodilation, increased arterial stiffness, and dysregulation of systemic metabolic-vascular coupling mechanisms [46].

Based on the pathophysiological traits of CKM syndrome, excessive adipose tissue hyperplasia and dysfunction drive the activation of a systemic oxidative stress-inflammatory axis (ROS/RNS-proinflammatory cytokine network), leading to endothelial insulin resistance, dysregulation of vascular smooth muscle calcium homeostasis, and extracellular matrix (ECM) fibrotic remodeling. These processes establish the pathophysiological basis for metabolic-vascular coupling dysfunction [17,46]. Upon inhalation, air pollutant particles exacerbate oxidative stress burden (elevated circulating ROS levels) and trigger the release of proinflammatory cytokines (e.g., TNF-α, IL-6) through mechanisms such as NLRP3 inflammasome and NF-κB pathway activation, thereby inducing phenotypic transitions in vascular endothelial cells and macrophages. The synergistic interplay between these cell types along the oxidative stress-inflammatory axis lowers the oxidative stress threshold, hyperactivates the cytokine network, and depletes compensatory vasodilatory reserves in CKM syndrome patients. This pathological triad ultimately manifests as an elevated risk of cardiovascular events.

The strengths of this study are as follows: First, leveraging a nationwide cohort design with a substantial temporal span and large sample size, it provides robust data support for elucidating long-term health effects. Second, air pollution exposure was quantified using high-resolution monitoring technologies, ensuring data quality and precision to accurately assess individual-level exposures, thereby laying a solid foundation for subsequent mechanistic analyses and risk evaluations. Third, as the first systematic investigation of the CKM syndrome cohort, it fills a critical research gap and offers pioneering theoretical evidence for guiding clinical interventions and public health strategies. Fourth, the study employed an innovative dual-dimensional analytical framework integrating single-pollutant and mixture exposure models, comprehensively capturing both independent and synergistic effects of pollutant exposures to support the construction of refined exposure-response models.

We observed that in this study, there was no significant statistical association between O₃ and the risk of CVD, which shows a certain discrepancy from previous studies. Two recent cohort studies from China indicated that long-term exposure to O₃ is positively correlated with the incidence of cardiovascular diseases, especially showing a notable manifestation in heart diseases and hypertension [10].This discrepancy may be closely related to differences in the study regions, time scales, as well as monitoring and analytical methods. High concentrations of nitrogen oxides (NOx) emitted from traffic and industrial sources can trigger the “titration effect” [47], where NO reacts with O₃ to form NO₂, effectively suppressing the ground-level O₃ concentration. Therefore, in high-NOx environments, the O₃ level may be below the threshold associated with CVD-related outcomes.From the perspective of time scales, some studies have focused on short-term, high-concentration ozone pollution events. During these specific periods, the sharp rise in ozone concentration makes it dominant in air quality changes. In contrast, this study conducted a comprehensive assessment over a longer time period. After long-term averaging, the contribution of ozone may be diluted by the persistent impact of other pollutants (such as particulate matter), thus appearing relatively small.Additionally, different monitoring methods vary in terms of measurement accuracy and range for ozone concentration. Different settings of input parameters in model simulations can also significantly influence the assessment results of ozone’s contribution. Finally, some studies have found that O₃ can reduce pro-inflammatory cytokines and activate the IL-10 anti-inflammatory cytokine. Given that part of the mechanism by which patients with CKM syndrome progress to clinical CVD involves the excessive activation of the inflammatory cytokine network [48], it can be inferred that moderate O₃ levels may not significantly increase the risk of clinical CVD in patients at CKM stages 0–3. In summary, there is still controversy over whether O₃ can promote the occurrence of clinical CVD in patients at CKM stages 0–3, and more research is needed.

This study confirms that air pollutants (particularly PM₁₀) accelerate cardiovascular disease progression in patients with CKM syndrome, with significant synergistic effects from multi-pollutant co-exposure, while O₃ demonstrates weaker associations. Based on these findings, we recommend designating PM₁₀ as a core indicator for CKM risk stratification and developing tiered prevention strategies tailored to varying exposure levels (e.g., intensified statin prophylaxis in high-pollution areas, seasonal vitamin D supplementation). Simultaneously, we advocate for integrating environmental policies with clinical management through electronic health systems embedded with air pollution alerts to enable precision interventions, thereby providing an actionable public health framework for CKM prevention and control in polluted regions.

In regions with severe environmental pollution, patients with CKM syndrome who cannot achieve immediate environmental remediation may benefit from lifestyle modifications. These include promoting regular physical activity, implementing weight-loss interventions for obese/overweight individuals, and reducing dietary intake of carbohydrates and fats. Such measures have been shown to decrease oxidative stress, alleviate insulin resistance, and mitigate metabolic risk factors. Epidemiological studies demonstrate that individuals with elevated body mass index (BMI) during follow-up exhibit significantly higher incidence rates of impaired glucose tolerance and type 2 diabetes mellitus compared to those with stable or reduced BMI [49].

Limitations of this study include the following: First, disease diagnoses primarily relied on self-reported data from participants, which may introduce information distortion due to individual cognitive differences or recall bias, thereby compromising the objectivity of study findings. Second, despite the high resolution of air pollution monitoring data, discrepancies may exist between actual individual exposures and measured values due to heterogeneous pollutant emission sources, complex atmospheric dispersion/dilution processes, and dynamic physicochemical transformations. These biases necessitate further calibration incorporating individual behavioral patterns and microenvironmental characteristics. Third, due to data constraints, we were unable to evaluate the contributions of additional potential exposure differentials—including rural-urban disparities, participant mobility, and indoor/outdoor exposure variations—which may affect the generalizability of our findings. Fourth, constrained by the variable collection scope of the CHARLS database, certain potential confounders (e.g., occupational exposures, lifestyle factors) were excluded from the analytical framework, potentially confounding causal inferences. Future studies should optimize research designs through multi-source data integration or supplementary surveys to address these limitations.

## Supporting information

S1 Table Definitions of CKM Syndrome Stages. (DOCX)

S1 Fig Pearson correlation between five types of air pollutants. (TIFF)

S2 Fig The association between five air pollutants and the risk of cardiovascular disease estimated by BKMR model. (PDF)

S3 Fig The association between air pollutant mixtures and the risk of cardiovascular disease. (TIFF)

S2 Table Cox regression analysis after excluding CVD patients who developed symptoms within 2 years after exposure assessment. (DOCX)

S3 Table Cox regression analysis after adding confounding variables to Model 3. (DOCX)

## References

[1] Zhou M, Wang H, Zeng X, Yin P, Zhu J, Chen W, et al. Mortality, morbidity, and risk factors in China and its provinces, 1990-2017: a systematic analysis for the Global Burden of Disease Study 2017. Lancet. 2019;394(10204):1145–58. doi: 10.1016/S0140-6736(19)30427-1 31248666 PMC6891889

[2] Brauer M, Roth GA, Aravkin AY, Zheng P, Abate KH, Abate YH, et al. Global burden and strength of evidence for 88 risk factors in 204 countries and 811 subnational locations, 1990–2021: a systematic analysis for the Global Burden of Disease Study 2021. The Lancet. 2024;403(10440):2162–203. 38762324 10.1016/S0140-6736(24)00933-4 PMC11120204

[3] Cole MP, Freeman BA. Promotion of cardiovascular disease by exposure to the air pollutant ozone. Am J Physiol Lung Cell Mol Physiol. 2009;297(2):L205-8. doi: 10.1152/ajplung.00187.2009 19525390 PMC2742797

[4] Wen F, Li B, Cao H, Li P, Xie Y, Zhang F, et al. Association of long-term exposure to air pollutant mixture and incident cardiovascular disease in a highly polluted region of China. Environ Pollut. 2023;328:121647. doi: 10.1016/j.envpol.2023.121647 37062405

[5] Liu Q, Pan L, He H, Hu Y, Tu J, Zhang L, et al. Effects of long-term exposure to air pollutant mixture on blood pressure in typical areas of North China. Ecotoxicol Environ Saf. 2024;285:116987. doi: 10.1016/j.ecoenv.2024.116987 39299210

[6] Li Z-H, Wang X-M, Liao D-Q, Zhang Q, Chen Z-T, Qiu C-S, et al. Long-term air pollutants exposure and respiratory mortality: A large prospective cohort study. Ecotoxicol Environ Saf. 2024;274:116176. doi: 10.1016/j.ecoenv.2024.116176 38479309

[7] Li Z, Liu Q, Chen L, Zhou L, Qi W, Wang C, et al. Ambient air pollution contributed to pulmonary tuberculosis in China. Emerg Microbes Infect. 2024;13(1):2399275. doi: 10.1080/22221751.2024.2399275 39206812 PMC11378674

[8] Wang KC, Lo YC, Liao CC, Jou YY, Huang HB. Associations between symptoms of depression and air pollutant exposure among older adults: results from the Taiwan longitudinal study on aging (TLSA). Frontiers in Public Health. 2021;9:779192. 35096739 10.3389/fpubh.2021.779192 PMC8790292

[9] King JD, Zhang S, Cohen A. Air pollution and mental health: associations, mechanisms and methods. Curr Opin Psychiatry. 2022;35(3):192–9. doi: 10.1097/YCO.0000000000000771 35579873

[10] Liang S, Chen Y, Sun X, Dong X, He G, Pu Y, et al. Long-term exposure to ambient ozone and cardiovascular diseases: Evidence from two national cohort studies in China. J Adv Res. 2024;62:165–73. doi: 10.1016/j.jare.2023.08.010 37625570 PMC11331174

[11] Gao H, Li J, Ma Q, Zhang Q, Li M, Hu X. Causal Associations of Environmental Pollution and Cardiovascular Disease: A Two-Sample Mendelian Randomization Study. Glob Heart. 2024;19(1):52. doi: 10.5334/gh.1331 38911616 PMC11192098

[12] Miller MR, Shaw CA, Langrish JP. From particles to patients: oxidative stress and the cardiovascular effects of air pollution. Future Cardiol. 2012;8(4):577–602. doi: 10.2217/fca.12.43 22871197

[13] Shaw CA, Robertson S, Miller MR, Duffin R, Tabor CM, Donaldson K, et al. Diesel exhaust particulate--exposed macrophages cause marked endothelial cell activation. Am J Respir Cell Mol Biol. 2011;44(6):840–51. doi: 10.1165/rcmb.2010-0011OC 20693402

[14] Donaldson K, Mills N, MacNee W, Robinson S, Newby D. Role of inflammation in cardiopulmonary health effects of PM. Toxicol Appl Pharmacol. 2005;207(2 Suppl):483–8. doi: 10.1016/j.taap.2005.02.020 15979665

[15] Mills NL, Törnqvist H, Robinson SD, Gonzalez M, Darnley K, MacNee W. Diesel exhaust inhalation causes vascular dysfunction and impaired endogenous fibrinolysis. Environ Health Perspect. 2005;112(25):3930–6. 10.1161/CIRCULATIONAHA.105.588962 16365212

[16] Robertson S, Miller MR. Ambient air pollution and thrombosis. Part Fibre Toxicol. 2018;15(1):1. doi: 10.1186/s12989-017-0237-x 29298690 PMC5753450

[17] Ndumele CE, Rangaswami J, Chow SL, Neeland IJ, Tuttle KR, Khan SS, et al. Cardiovascular-Kidney-Metabolic Health: A Presidential Advisory From the American Heart Association. Circulation. 2023;148(20):1606–35. doi: 10.1161/CIR.0000000000001184 37807924

[18] Li N, Li Y, Cui L, Shu R, Song H, Wang J, et al. Association between different stages of cardiovascular-kidney-metabolic syndrome and the risk of all-cause mortality. Atherosclerosis. 2024;397:118585. doi: 10.1016/j.atherosclerosis.2024.118585 39255681

[19] Cesaro A, Acerbo V, Scialla F, Golia E, Concilio C, Scherillo G. Discrepancies between physician-perceived and calculated cardiovascular risk in primary prevention: implications for LDL-C target achievement and appropriate lipid-lowering therapy. High blood pressure & cardiovascular prevention: the official journal of the Italian Society of Hypertension. 2025;32(2):199–208. 39969794 10.1007/s40292-025-00705-0 PMC11890243

[20] Li W, Shen C, Kong W, Zhou X, Fan H, Zhang Y, et al. Association between the triglyceride glucose-body mass index and future cardiovascular disease risk in a population with Cardiovascular-Kidney-Metabolic syndrome stage 0-3: a nationwide prospective cohort study. Cardiovasc Diabetol. 2024;23(1):292. doi: 10.1186/s12933-024-02352-6 39113004 PMC11308445

[21] Hu Y, Liang Y, Li J, Li X, Yu M, Cui W. Correlation between atherogenic index of plasma and cardiovascular disease risk across Cardiovascular-kidney-metabolic syndrome stages 0-3: a nationwide prospective cohort study. Cardiovasc Diabetol. 2025;24(1):40. doi: 10.1186/s12933-025-02593-z 39856691 PMC11763136

[22] Liang X, Lai K, Li X, Gui S, Xing Z, Li Y. U-shaped relationship of estimated glucose disposal rate with cardiovascular disease risk in cardiovascular-kidney-metabolic syndrome stages 0-3: a population-based prospective study. Diabetol Metab Syndr. 2025;17(1):85. doi: 10.1186/s13098-025-01659-y 40069902 PMC11895221

[23] Shen X, Liu Q, Lin T, Zheng D, He Q. Association between Chinese visceral adiposity index and cardiovascular events risk in individuals with cardiovascular-kidney-metabolic syndrome stage 0-3: a nationwide cohort study. Int Urol Nephrol. 2025;57(7):2255–69. doi: 10.1007/s11255-025-04403-7 39934555

[24] Shang Z, Feng S-T, Qian H, Deng Z-L, Wang Y, Gao Y-M. The impact of the triglyceride-glucose index on the deterioration of kidney function in patients with cardiovascular-kidney-metabolic syndrome: insight from a large cohort study in China. Ren Fail. 2025;47(1):2446656. doi: 10.1080/0886022X.2024.2446656 39757592 PMC11721749

[25] Zhao Y, Hu Y, Smith JP, Strauss J, Yang G. Cohort profile: the China Health and Retirement Longitudinal Study (CHARLS). Int J Epidemiol. 2014;43(1):61–8. doi: 10.1093/ije/dys203 23243115 PMC3937970

[26] D’Agostino RB Sr, Vasan RS, Pencina MJ, Wolf PA, Cobain M, Massaro JM, et al. General Cardiovascular Risk Profile for Use in Primary Care. Circulation. 2008;117(6):743–53. doi: 10.1161/circulationaha.107.699579 18212285

[27] Ma Y-C, Zuo L, Chen J-H, Luo Q, Yu X-Q, Li Y, et al. Modified glomerular filtration rate estimating equation for Chinese patients with chronic kidney disease. J Am Soc Nephrol. 2006;17(10):2937–44. doi: 10.1681/ASN.2006040368 16988059

[28] Wei J, Li Z, Lyapustin A, Sun L, Peng Y, Xue W, et al. Reconstructing 1-km-resolution high-quality PM2.5 data records from 2000 to 2018 in China: spatiotemporal variations and policy implications. Remote Sensing of Environment. 2021;252:112136. doi: 10.1016/j.rse.2020.112136

[29] Huang L, Hu X, Liu J, Wang J, Zhou Y, Li G, et al. Air pollution is linked to cognitive decline independent of hypersensitive C-reactive protein: insights from middle-aged and older Chinese. Environ Health. 2024;23(1):111. doi: 10.1186/s12940-024-01148-1 39707297 PMC11662488

[30] Dong Y, Cao W, Wei J, Chen Y, Zhang Y, Sun S, et al. Health effect of multiple air pollutant mixture on sarcopenia among middle-aged and older adults in China. Ecotoxicol Environ Saf. 2024;281:116634. doi: 10.1016/j.ecoenv.2024.116634 38925034

[31] Chen Y, Xu Z, Guo Y, Li S, Wang YA, Gasevic D. Air pollution increases the risk of frailty: China Health and Retirement Longitudinal Study (CHARLS). J Hazard Mater. 2025;492:138105. doi: 10.1016/j.jhazmat.2025.138105 40187242

[32] Gao K, Cao L-F, Ma W-Z, Gao Y-J, Luo M-S, Zhu J, et al. Association between sarcopenia and cardiovascular disease among middle-aged and older adults: Findings from the China health and retirement longitudinal study. EClinicalMedicine. 2022;44:101264. doi: 10.1016/j.eclinm.2021.101264 35059617 PMC8760427

[33] Zhang X, Ding L, Hu H, He H, Xiong Z, Zhu X. Associations of Body-Roundness Index and Sarcopenia with Cardiovascular Disease among Middle-Aged and Older Adults: Findings from CHARLS. J Nutr Health Aging. 2023;27(11):953–9. doi: 10.1007/s12603-023-2001-2 37997715 PMC12876593

[34] Li F, Wang Y, Shi B, Sun S, Wang S, Pang S, et al. Association between the cumulative average triglyceride glucose-body mass index and cardiovascular disease incidence among the middle-aged and older population: a prospective nationwide cohort study in China. Cardiovasc Diabetol. 2024;23(1):16. doi: 10.1186/s12933-023-02114-w 38184577 PMC10771655

[35] Gennings C, Curtin P, Bello G, Wright R, Arora M, Austin C. Lagged WQS regression for mixtures with many components. Environ Res. 2020;186:109529. doi: 10.1016/j.envres.2020.109529 32371274 PMC7489300

[36] Keil AP, Buckley JP, O’Brien KM, Ferguson KK, Zhao S, White AJ. A Quantile-Based g-Computation Approach to Addressing the Effects of Exposure Mixtures. Environ Health Perspect. 2020;128(4):47004. doi: 10.1289/EHP5838 32255670 PMC7228100

[37] Bobb JF, Valeri L, Claus Henn B, Christiani DC, Wright RO, Mazumdar M, et al. Bayesian kernel machine regression for estimating the health effects of multi-pollutant mixtures. Biostatistics. 2015;16(3):493–508. doi: 10.1093/biostatistics/kxu058 25532525 PMC5963470

[38] Brook RD, Rajagopalan S, Pope CA, Brook JR, Bhatnagar A, Diez-Roux AV. Particulate Matter Air Pollution and Cardiovascular Disease. 2010;121(21):2331–78. 10.1161/CIR.0b013e3181dbece1 20458016

[39] Fujimaki H, Kurokawa Y, Yamamoto S, Satoh M. Distinct requirements for interleukin-6 in airway inflammation induced by diesel exhaust in mice. Immunopharmacol Immunotoxicol. 2006;28(4):703–14. doi: 10.1080/08923970601067433 17190745

[40] Seaton A, MacNee W, Donaldson K, Godden D. Particulate air pollution and acute health effects. Lancet. 1995;345(8943):176–8. doi: 10.1016/s0140-6736(95)90173-6 7741860

[41] Furuyama A, Kanno S, Kobayashi T, Hirano S. Extrapulmonary translocation of intratracheally instilled fine and ultrafine particles via direct and alveolar macrophage-associated routes. Arch Toxicol. 2009;83(5):429–37. doi: 10.1007/s00204-008-0371-1 18953527

[42] Sørensen M, Daneshvar B, Hansen M, Dragsted LO, Hertel O, Knudsen L, et al. Personal PM2.5 exposure and markers of oxidative stress in blood. Environ Health Perspect. 2003;111(2):161–6. doi: 10.1289/ehp.111-1241344 12573899 PMC1241344

[43] Pekkanen J, Brunner EJ, Anderson HR, Tiittanen P, Atkinson RW. Daily concentrations of air pollution and plasma fibrinogen in London. 2000;57(12):818–22. 10.1136/oem.57.12.818 PMC1739901 11077010

[44] Eringa EC, Bakker W, Smulders YM, Serné EH, Yudkin JS, Stehouwer CDA. Regulation of vascular function and insulin sensitivity by adipose tissue: focus on perivascular adipose tissue. Microcirculation. 2007;14(4–5):389–402. doi: 10.1080/10739680701303584 17613810

[45] Saxton SN, Clark BJ, Withers SB, Eringa EC, Heagerty AM. Mechanistic Links Between Obesity, Diabetes, and Blood Pressure: Role of Perivascular Adipose Tissue. Physiol Rev. 2019;99(4):1701–63. doi: 10.1152/physrev.00034.2018 31339053

[46] Padilla J, Vieira-Potter VJ, Jia G, Sowers JR. Role of perivascular adipose tissue on vascular reactive oxygen species in type 2 diabetes: a give-and-take relationship. Diabetes. 2015;64(6):1904–6. doi: 10.2337/db15-0096 25999534 PMC4439571

[47] Wang T, Xue L, Brimblecombe P, Lam YF, Li L, Zhang L. Ozone pollution in China: A review of concentrations, meteorological influences, chemical precursors, and effects. Sci Total Environ. 2017;575:1582–96. doi: 10.1016/j.scitotenv.2016.10.081 27789078

[48] Tartari APS, Moreira FF, Pereira MCDS, Carraro E, Cidral-Filho FJ, Salgado AI, et al. Anti-inflammatory Effect of Ozone Therapy in an Experimental Model of Rheumatoid Arthritis. Inflammation. 2020;43(3):985–93. doi: 10.1007/s10753-020-01184-2 32382842

[49] Wilson PW, Kannel WB, Silbershatz H, D’Agostino RB. Clustering of metabolic factors and coronary heart disease. Arch Intern Med. 1999;159(10):1104–9. doi: 10.1001/archinte.159.10.1104 10335688

