# Supplementary material for: The individual and combined effects of air pollution mixtures on the risk of cardiovascular diseases in patients with Cardiovascular-Kidney-Metabolic syndrome at stages 0–3
Source: PLoS One. 2026 Jun 26;21(6):e0346949. doi: 10.1371/journal.pone.0346949 (PMC13308838; doi:10.1371/journal.pone.0346949)
Supplement: S1 Table — (DOCX) [file pone.0346949.s001.docx]

**S1 Table. Definitions of CKM Syndrome Stages**

| CKM syndrome stages | Definition |
| --- | --- |
| Stage 0 | Individuals show no evidence of metabolic abnormalities, cardiovascular lesions, or renal impairment |
| Stage 1 | Features of this stage include overweight/obesity (with a BMI ≥ 25 kg/m² or excessive waist circumference) or isolated adipose tissue dysfunction (without accompanying metabolic risk factors such as hypertension, dyslipidemia, or chronic kidney disease [CKD]) |
| Stage 2 | Individuals with metabolic risk factors: hypertriglyceridemia (≥135 mg/dL), hypertension (≥130/80 mmHg), metabolic syndrome, diabetes mellitus, or a confirmed diagnosis of CKD (defined by an estimated glomerular filtration rate [eGFR] < 60 mL/min/1.73m², calculated using the Chinese-modified Modification of Diet in Renal Disease [C-MDRD] equation) |
| Stage 3 | Subclinical ASCVD or subclinical HF (indicated by a 10-year cardiovascular event risk ≥ 10% as predicted by the Framingham Risk Score, or CKD progression to stages G4-G5 (eGFR < 30 mL/min/1.73m²) and classified as very high risk according to the Kidney Disease: Improving Global Outcomes (KDIGO) guidelines. The eGFR is determined using the C-MDRD equation (28), and is employed to categorize CKD stages in line with KDIGO criteria.) in patients with excessive/dysfunctional obesity, other metabolic risk factors, or CKD |
| Stage 4 | Clinical CVD (coronary artery disease, HF, stroke, peripheral artery disease, atrial fibrillation) in individuals with excessive/dysfunctional obesity, other CKM risk factors, or CKD.  4a：Without renal failure  4b：With renal failure |
