# Supplementary figures and images for: The individual and combined effects of air pollution mixtures on the risk of cardiovascular diseases in patients with Cardiovascular-Kidney-Metabolic syndrome at stages 0–3
Source: PLoS One. 2026 Jun 26;21(6):e0346949. doi: 10.1371/journal.pone.0346949 (PMC13308838; doi:10.1371/journal.pone.0346949)

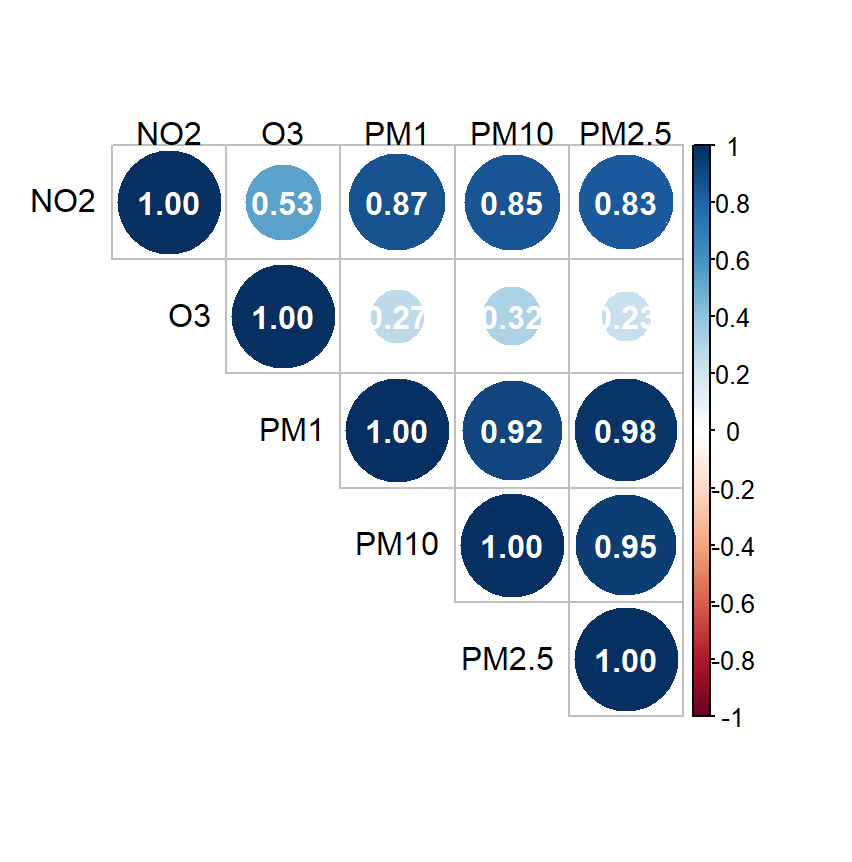

Supplement: S1 Fig — (TIFF) [file pone.0346949.s002.tiff]

**A**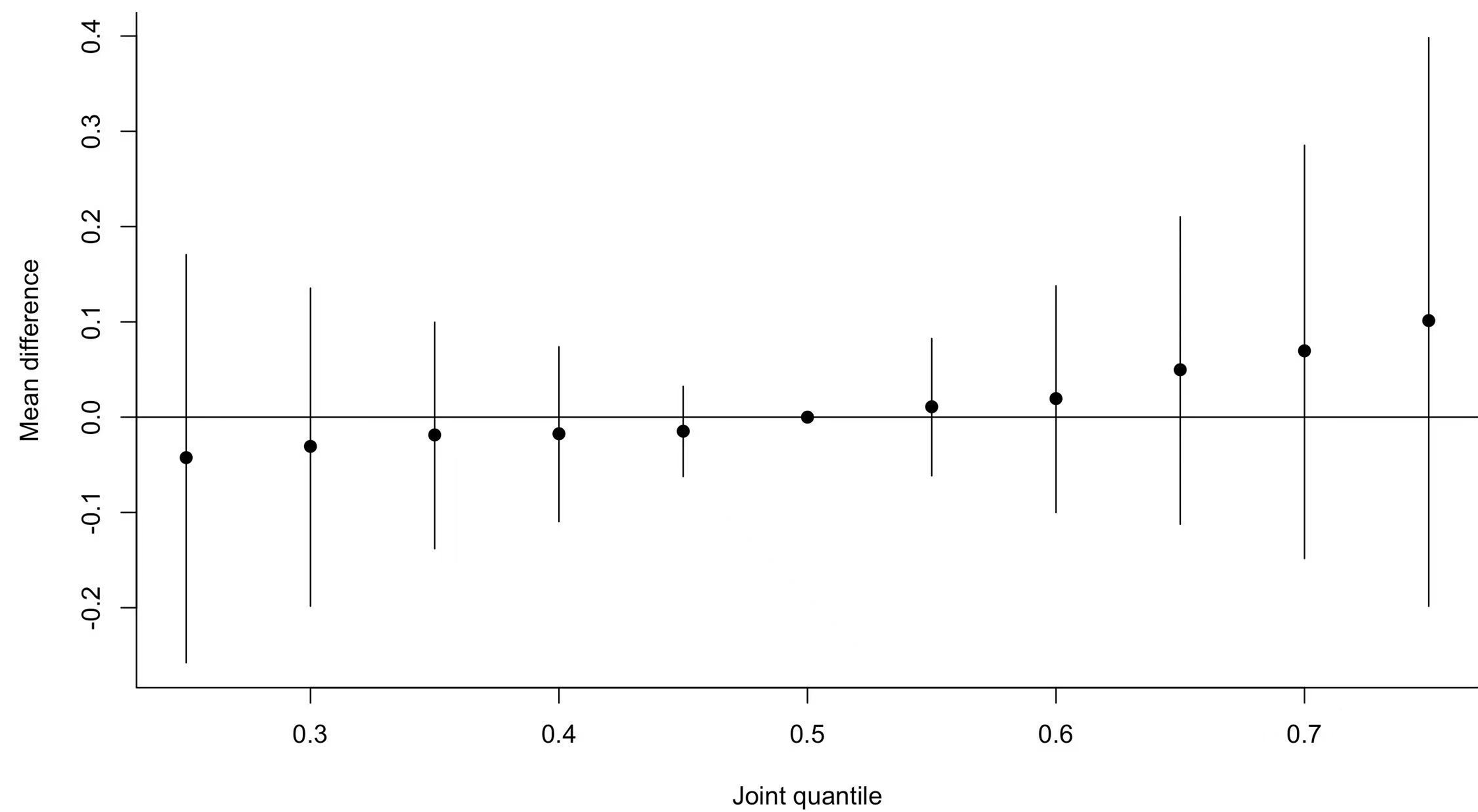**B**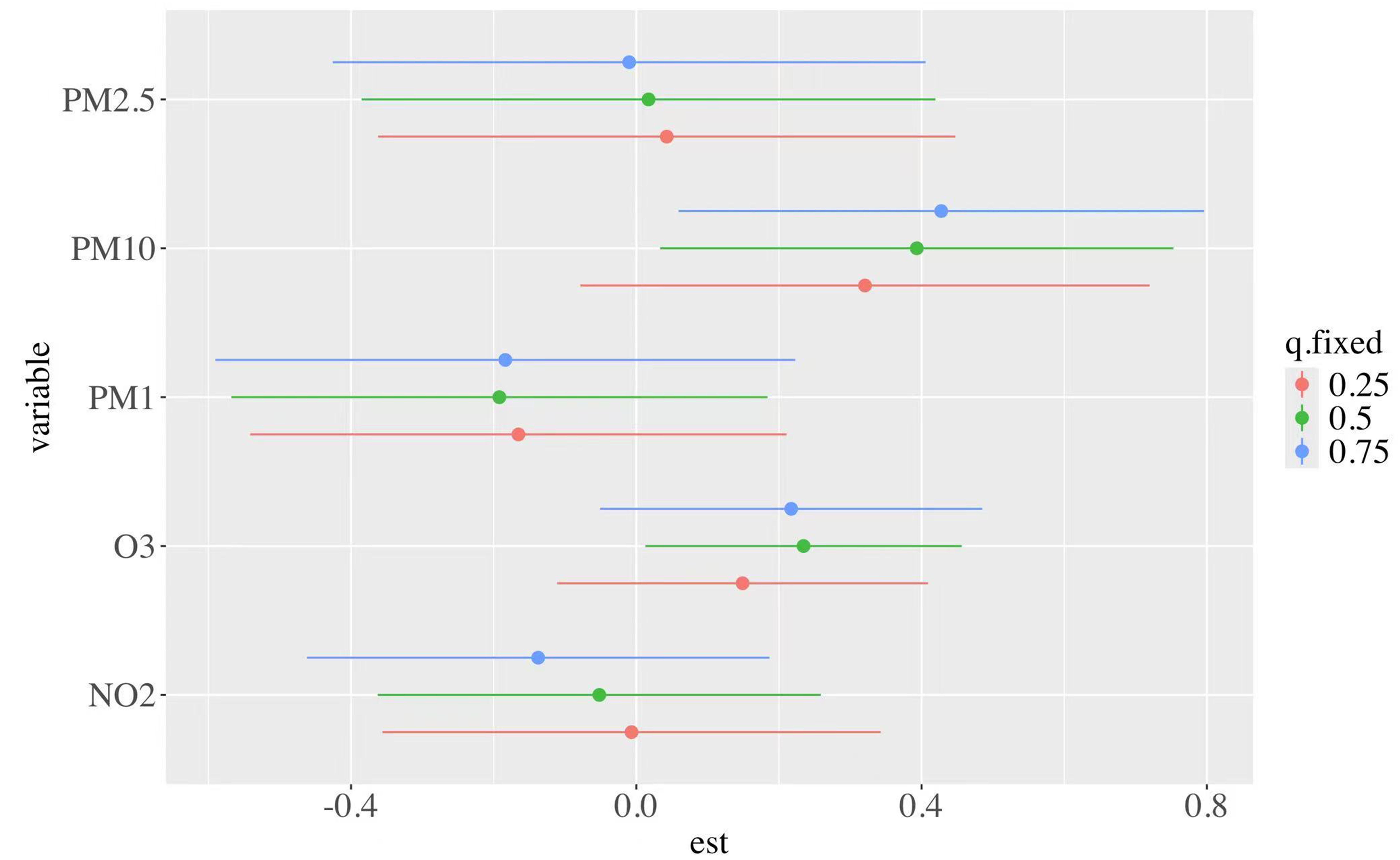

Supplement: S2 Fig — (PDF) [file pone.0346949.s003.pdf]

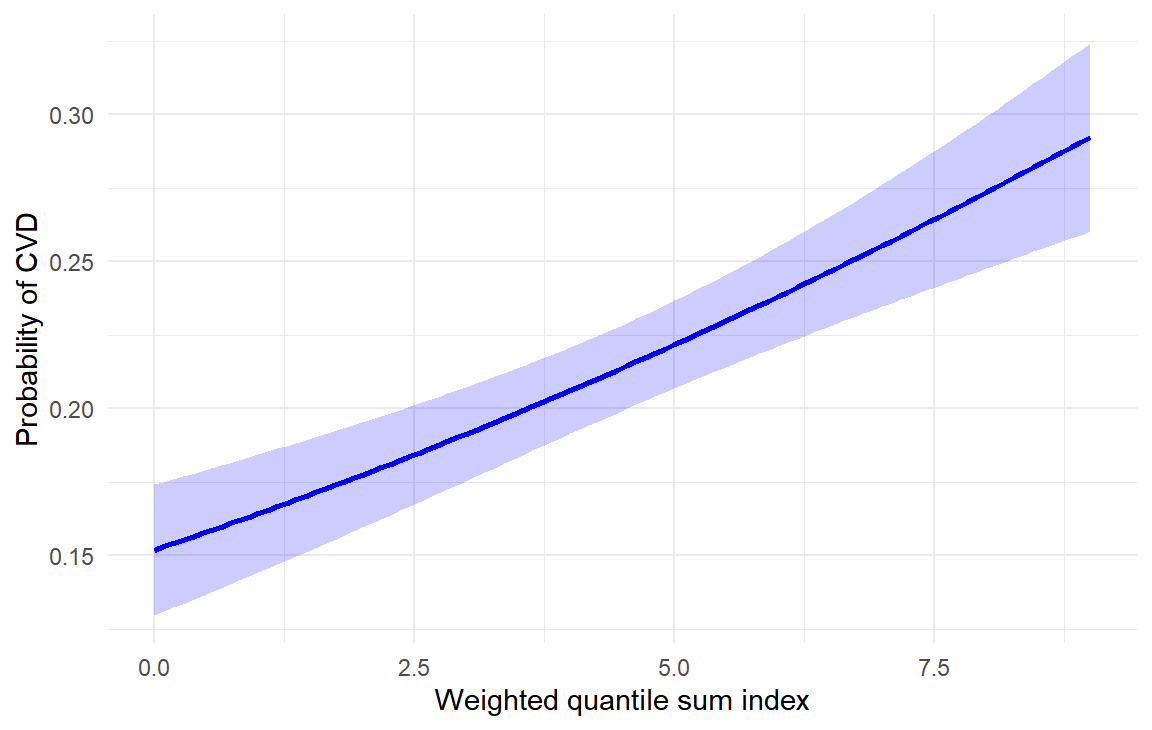

Supplement: S3 Fig — (TIFF) [file pone.0346949.s004.tiff]
