# Supplementary material for: The individual and combined effects of air pollution mixtures on the risk of cardiovascular diseases in patients with Cardiovascular-Kidney-Metabolic syndrome at stages 0–3
Source: PLoS One. 2026 Jun 26;21(6):e0346949. doi: 10.1371/journal.pone.0346949 (PMC13308838; doi:10.1371/journal.pone.0346949)
Supplement: S2 Table — (DOCX) [file pone.0346949.s005.docx]

**S2 Table. Cox regression analysis after excluding CVD patients who developed symptoms within 2 years after exposure assessment**

| Air pollutant  (IQR) | Model 1^a^ | | Model 2^b^ | | Model 3^c^ | |
| --- | --- | --- | --- | --- | --- | --- |
|  | HR(95%CI) | P value | HR (95 % CI) | P value | HR (95 % CI) | P value |
| NO2 | 1.26(1.13,1.41) | <0.001 | 1.28(1.14,1.44) | <0.001 | 1.30(1.16,1.47) | <0.001 |
| O3 | 0.98(0.89,1.08) | 0.680 | 0.99(0.90,1.08) | 0.770 | 1.00(0.90,1.10) | 0.928 |
| PM1 | 1.31(1.17,1.46) | <0.001 | 1.32(1.18,1.48) | <0.001 | 1.34(1.19,1.50) | <0.001 |
| PM2.5 | 1.36(1.21,1.52) | <0.001 | 1.36(1.21,1.54) | <0.001 | 1.38(1.22,1.55) | <0.001 |
| PM10 | 1.49(1.33,1.68) | <0.001 | 1.53(1.35,1.73) | <0.001 | 1.54(1.36,1.74) | <0.001 |

^a^Model 1 represented the unadjusted crude model.

^b^Model 2 adjusted for sociodemographic characteristics (including age, gender, place of residence, educational attainment, marital status, total per capita household consumption, and type of cooking fuel).

^c^Model 3 further incorporated behavioral health factors (smoking status, alcohol consumption, and sleep disorders) on top of the variables in Model 2
