# Supplementary material for: The individual and combined effects of air pollution mixtures on the risk of cardiovascular diseases in patients with Cardiovascular-Kidney-Metabolic syndrome at stages 0–3
Source: PLoS One. 2026 Jun 26;21(6):e0346949. doi: 10.1371/journal.pone.0346949 (PMC13308838; doi:10.1371/journal.pone.0346949)
Supplement: S3 Table — (DOCX) [file pone.0346949.s006.docx]

**S3 Table. Cox regression analysis after adding confounding variables to Model 3**

| Air pollutant  (IQR) | Model 1^a^ | | Model 2^b^ | | Model 3^c^ | |
| --- | --- | --- | --- | --- | --- | --- |
|  | HR(95%CI) | P value | HR (95 % CI) | P value | HR (95 % CI) | P value |
| NO_2_(15.1μg/m³) | 1.26(1.13,1.39) | <0.001 | 1.28(1.15,1.43) | <0.001 | 1.20(1.07,1.35) | 0.001 |
| O_3_(7.5μg/m³) | 0.97(0.89,1.06) | 0.500 | 0.98(0.90,1.07) | 0.622 | 0.95(0.86,1.04) | 0.231 |
| PM_1_(14.1μg/m³) | 1.28(1.15,1.41) | <0.001 | 1.28(1.16,1.43) | <0.001 | 1.24(1.11,1.38) | <0.001 |
| PM_2.5_(27.4μg/m³) | 1.33(1.19,1.47) | <0.001 | 1.33(1.19,1.48) | <0.001 | 1.27(1.14,1.43) | <0.001 |
| PM_10_(48.7μg/m³) | 1.47(1.32,1.64) | <0.001 | 1.50(1.34,1.68) | <0.001 | 1.43(1.27,1.61) | <0.001 |

^a^Model 1 represented the unadjusted crude model.

^b^Model 2 adjusted for sociodemographic characteristics (including age, gender, place of residence, educational attainment, marital status, total per capita household consumption, and type of cooking fuel).

^c^Model 3 further incorporated behavioral health factors (smoking status, alcohol consumption, and sleep disorders) on top of the variables in Model 2
